# Investigation of Supported Pd-Based Electrocatalysts for the Oxygen Reduction Reaction: Performance, Durability and Methanol Tolerance

**DOI:** 10.3390/ma8125438

**Published:** 2015-11-25

**Authors:** Carmelo Lo Vecchio, Cinthia Alegre, David Sebastián, Alessandro Stassi, Antonino S. Aricò, Vincenzo Baglio

**Affiliations:** CNR-ITAE Institute, Via Salita Santa Lucia sopra Contesse, 5, Messina 98126, Italy; lovecchio@itae.cnr.it (C.L.V.); alegre@itae.cnr.it (C.A.); sebastian@itae.cnr.it (D.S.); alessandro.stassi@itae.cnr.it (A.S.); arico@itae.cnr.it (A.S.A.)

**Keywords:** direct methanol fuel cells, oxygen reduction reaction, methanol tolerance, Pd catalysts, durability tests, titanium suboxides

## Abstract

Next generation cathode catalysts for direct methanol fuel cells (DMFCs) must have high catalytic activity for the oxygen reduction reaction (ORR), a lower cost than benchmark Pt catalysts, and high stability and high tolerance to permeated methanol. In this study, palladium catalysts supported on titanium suboxides (Pd/Ti*_n_*O_2*n*–1_) were prepared by the sulphite complex route. The aim was to improve methanol tolerance and lower the cost associated with the noble metal while enhancing the stability through the use of titanium-based support; 30% Pd/Ketjenblack (Pd/KB) and 30% Pd/Vulcan (Pd/Vul) were also synthesized for comparison, using the same methodology. The catalysts were *ex-situ* characterized by physico-chemical analysis and investigated for the ORR to evaluate their activity, stability, and methanol tolerance properties. The Pd/KB catalyst showed the highest activity towards the ORR in perchloric acid solution. All Pd-based catalysts showed suitable tolerance to methanol poisoning, leading to higher ORR activity than a benchmark Pt/C catalyst in the presence of low methanol concentration. Among them, the Pd/Ti*_n_*O_2*n*–1_ catalyst showed a very promising stability compared to carbon-supported Pd samples in an accelerated degradation test of 1000 potential cycles. These results indicate good perspectives for the application of Pd/Ti*_n_*O_2*n*–1_ catalysts in DMFC cathodes.

## 1. Introduction

Fuel cells, fed with H_2_ or organic fuels, are promising energy conversion devices with low environmental impact [1,2]. DMFCs are very appropriate for portable power applications (such as consumer electronics) where the power requirements are low and a simple compact system with high energy density is required [3,4].

The electrocatalysis of ORR is one of the limiting steps due to the slow kinetics. This requires the use of expensive Pt-based catalysts characterized by suitable activity. Moreover, one of the main drawbacks in DMFC is the methanol crossover from the anode to the cathode side through the membrane. Methanol permeation produces a mixed potential at the cathode, reducing the overall efficiency [5]. Thus, DMFC technology requires the development of a catalyst with lower cost and more suitable activity and selectivity towards ORR than Pt/C [6,7]. To achieve this objective, Pt-free catalysts with better methanol tolerance and proper activity are considered. In this regard, Pd-based electrocatalysts are promising candidates, since they are characterized by a low methanol poisoning [8,9,10].

Catalyst activity and stability also depend on structural and morphological features of the catalyst support [11]. Durability is an important issue that needs to be addressed in order to achieve a large-scale commercialization of proton exchange membrane fuel cells (PEMFCs) [12], of which DMFCs are a sub-category. In particular, cathode electrocatalyst degradation limits the durability of PEMFCs. The mechanisms causing a decrease of the catalyst (Pt, Pd, *etc.*) electrochemical surface area (ECSA) are summarized into the following points: (1) dissolution of metal ions from small particles into the membrane; (2) re-deposition onto larger particles; (3) corrosion of carbon support with loss of electronic contact or particle coalescence [13].

To improve the electrochemical activity and stability of the supported catalysts, significant work has been done to synthesize new carbon materials with controlled and tunable nanostructures [14,15,16,17,18]. However, the stability of carbon is not appropriate under some harsh operating conditions experienced by the cathode in a polymer electrolyte fuel cell [19]. These materials are prone to corrosion, especially at high electro-chemical potentials or in practical starvation conditions, leading to a fast and significant loss of ECSA over time during fuel cell operation [20]. With the aim of improving the cathode stability in DMFCs, a non-carbonaceous Ti-suboxide material was selected in the present work as a support for Pd electrocatalysts. Ti-suboxides are recognized as robust materials in aggressive media and are stable in harsh conditions such as those occurring at the cathode of PEMFCs [21]; moreover, they exhibit high electrical conductivity [22]. Ti-suboxides are also excellent supports for Ir-oxide anodes in PEM electrolyzers where electrochemical potentials as high as 2.0 V *vs.* reference hydrogen electrode (RHE) occur [23]. A comparison of the Pd/Ti*_n_*O_2*n*–1_ catalyst properties with respect to Pd supported on commonly used carbon blacks (Ketjenblack and Vulcan) is provided in terms of ORR activity, tolerance to methanol poisoning, and resistance to accelerated degradation tests. Also, an evaluation of the methanol tolerance properties of these Pd catalysts compared to a commercial Pt/C is presented.

## 2. Results and Discussion

### 2.1. XRD Analysis

**Figure 1:**
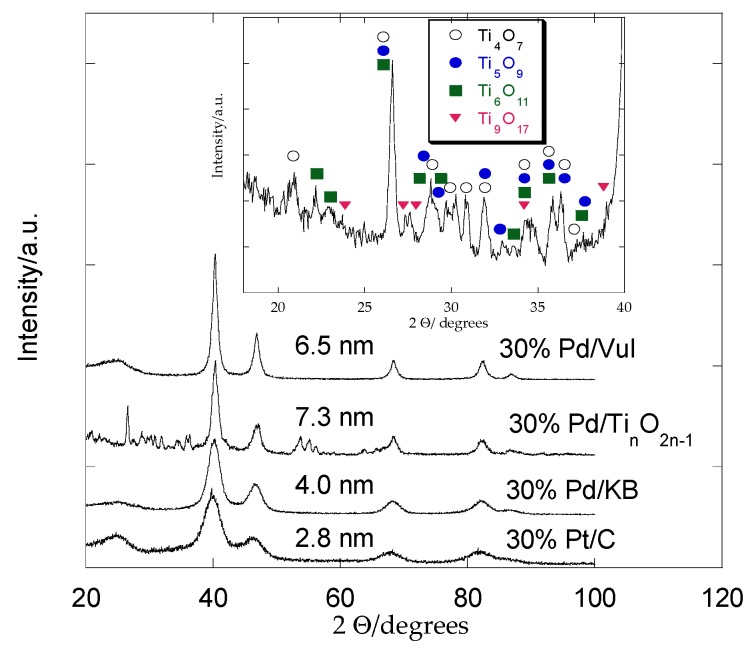
XRD patterns of 30% Pd/Vul, 30% Pd/KB, 30% Pd/Ti*_n_*O_2*n*__–__1_ and commercial 30% Pt/C. Inset: Ti-oxide phases for Pd/Ti*_n_*O_2*n*__–__1_ catalyst.

Figure 1 compares the XRD patterns of Pd catalysts dispersed on different supports and a commercial Pt catalyst on carbon. It can be seen that Pd/Vulcan, Pd/KB, Pd/Ti*_n_*O_2*n*–1_ and Pt/C show the typical face-centered cubic structure (fcc) of Pd and Pt. 

The C (002) graphite carbon reflection occurs at about 2θ = 25°. The typical peaks of Pd appear at 40.1°, 46.6°, 67.8° and 82.1° corresponding to (111), (200), (220) and (311) planes of the fcc structure of Pd, respectively. Regarding the 30% Pd/Ti*_n_*O_2*n*–1_, besides Pd reflections, some other peaks appear which are related to Ti-oxide phases such as Ti_4_O_7_, Ti_5_O_9_, Ti_6_O_11_ and Ti_9_O_17_ [23]. The main peaks of Ti_4_O_7_ (JCPDS: 18-1402), Ti_5_O_9_ (JCPDS: 11-193), Ti_6_O_11_ (JCPDS: 18-1401) and Ti_9_O_17_ (JCPDS: 18-1405) are indicated in the inset of Figure 1. The calculated crystallite size (by Scherrer’s equation) of Pd is 6.5 nm, 4 nm and 7.3 nm in Pd/Vul, Pd/KB and Pd/Ti*_n_*O_2*n*__–__1_, respectively, and 2.8 nm for Pt in Pt/C, referred to the 220 reflection peak at around 2θ = 68°. The presence of KB allows us to obtain a smaller crystallite size for the Pd-based catalysts due to the higher surface area (950 m^2^·g^−1^) of this carbon black compared to Vulcan (250 m^2^·g^−1^) [24]. The largest crystallite size for Pd (7.3 nm) is obtained with the support characterized by the lowest Brunauer, Emmett and Teller (BET) surface area (Ti*_n_*O_2*n*__–__1_, 2 m^2^·g^−1^). The commercial Pt/C shows the smallest crystallite size (2.8 nm).

### 2.2. TEM Analysis

**Figure 2:**
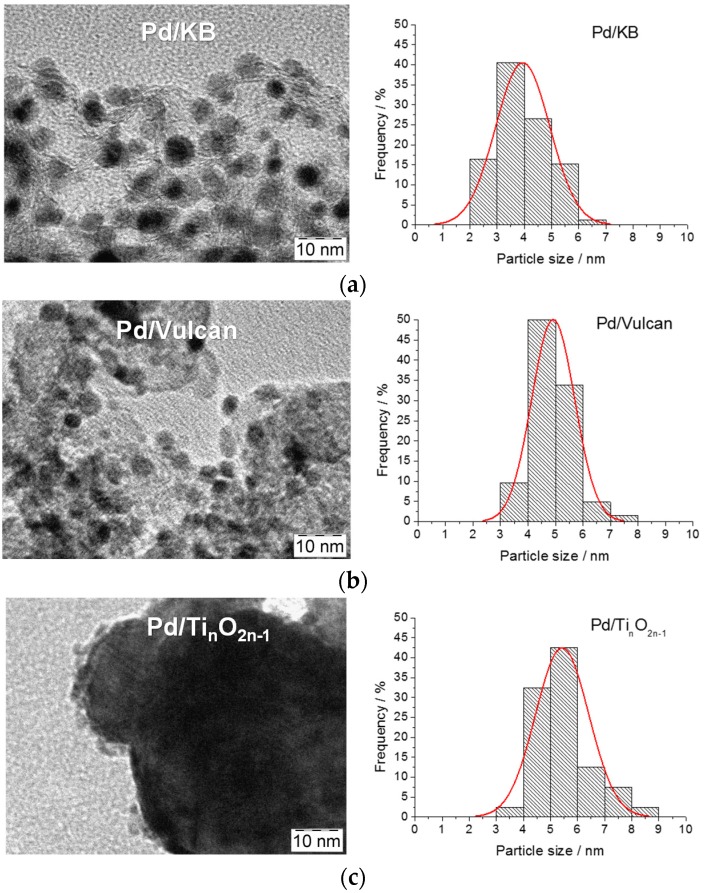
TEM micrographs for the Pd-based catalysts: (**a**) 30% Pd/KB; (**b**) 30% Pd/Vul; (**c**) 30% Pd/Ti*_n_*O_2*n*__–__1_.

TEM analysis (Figure 2) showed a good dispersion for the catalysts supported on carbon blacks (Vulcan and KB) and a mean particle size similar to the crystallite size determined by XRD (see histograms in Figure 2). For the catalyst supported on Ti-suboxides, the Pd nanoparticles are clearly visible only at the edges of the large support particles due to the low contrast between Ti and Pd species. Nonetheless, a suitable Pd particle distribution on the support is envisaged for all catalysts. Thermal gravimetric analysis confirmed a metal concentration (of 30 ± 2 wt %) for Pd/C electrocatalysts; the same result was obtained for the Pd/Ti*_n_*O_2*n*–1_ sample by means of EDX analysis.

### 2.3. Rotating Disk Electrode (RDE) Measurements

**Figure 3:**
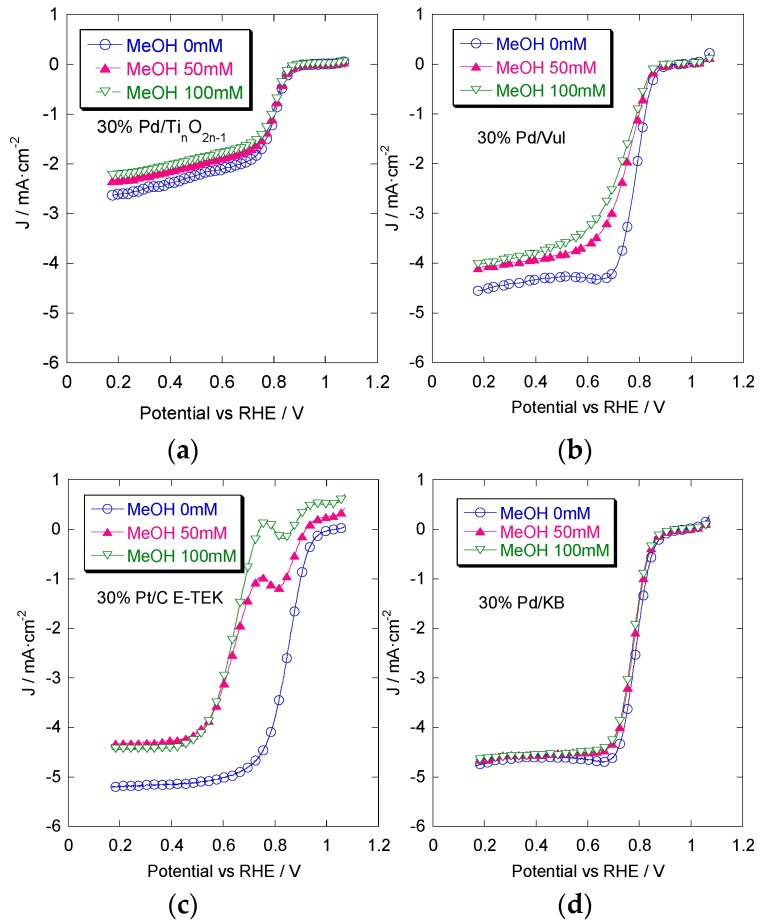
Linear sweep voltammetry of oxygen reduction at different MeOH concentrations (0; 50; 100 mM) for 30% Pt/C E-TEK (**a**); 30% Pd/KB (**b**); 30% Pd/Ti*_n_*O_2*n*__–__1_ (**c**) and 30% Pd/Vul (**d**). Scan rate 5 mV·s^−1^; 1000 rpm; room temperature and 50 μg·cm^−2^ of Pd or Pt loading.

Figure 3 shows the ORR curves obtained in O_2_-saturated perchloric acid without methanol and with two different methanol concentrations (50 mM; 100 mM). These curves allow us to evaluate the methanol tolerance of the different catalysts. The methanol addition causes a shift of the ORR onset towards more negative potential values and a decrease of current. This is due to the mixed potential caused by the simultaneous oxygen reduction and methanol oxidation at the working electrode interface. This effect is more remarkable in the commercial 30% Pt/C catalyst showing an evident methanol oxidation peak during the ORR experiment. The least pronounced shift is noted for the 30% Pd/KB catalyst with respect to the other Pd-supported catalysts. Such results confirm a better tolerance to methanol of the Pd-based catalysts.

The commercial 30% Pt/C catalyst shows the highest limiting current (i_l_) when the bare electrolyte (only 0.1 M HClO_4_) is used. The i_l_ decreases after the addition of methanol. The 30% Pd/KB has an excellent methanol tolerance and the limiting current is similar to the commercial Pt/C catalyst even in the presence of 100 mM methanol. Pd catalysts supported on Ti*_n_*O_2*n*–1_ and Vulcan both exhibit a good tolerance to methanol poisoning, although the best catalytic activity was obtained with KB as the support. These results point out the good methanol tolerance of Pd, whereas the support influences the level of catalytic activity according to the degree of catalyst dispersion.

**Figure 4:**
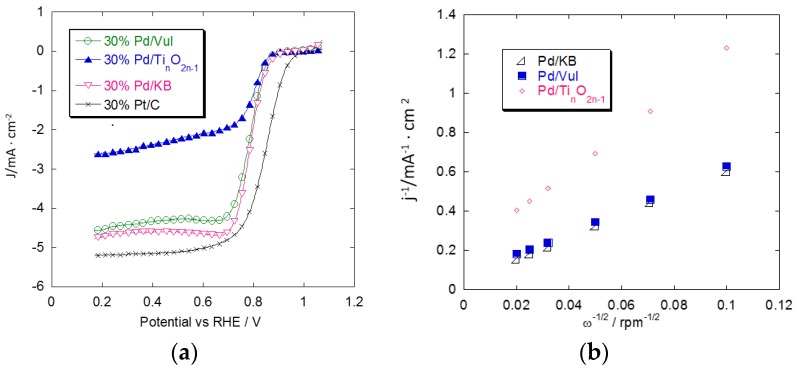
(**a**) Comparison of oxygen reduction voltammetric curves for the different Pd/support catalysts and the commercial Pt/C E-TEK catalyst. Scan rate 5 mV·s^−1^; 1000 rpm; room temperature and 50 μg·cm^−2^ of Pd or Pt loading; (**b**) Koutecky-Levich plots for 30% Pd/Vul, 30% Pd/KB, 30% Pd/Ti*_n_*O_2*n*__–__1_ obtained from the RDE data at 0.7 V *vs.* RHE.

**Figure 5:**
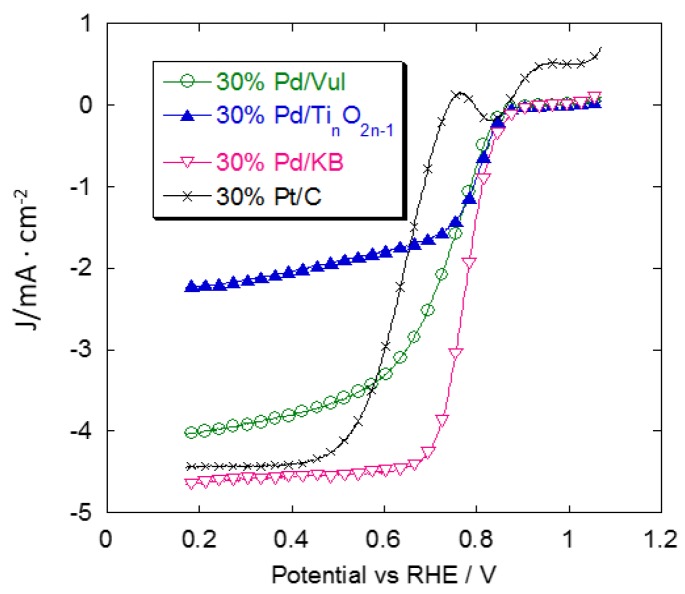
Comparison of oxygen reduction curves for the different Pd/support catalysts and the commercial Pt/C E-TEK catalyst in the presence of 0.1 M MeOH. Scan rate 5 mV·s^−1^; 1000 rpm; room temperature and 50 μg·cm^−2^ of Pd or Pt loading.

A comparison of ORR curves for all the above-mentioned catalysts is shown in Figure 4 and Figure 5. Figure 4a shows oxygen reduction curves obtained in the base electrolyte without methanol. The commercial 30% Pt/C has a higher activity than the Pd catalysts in terms of onset potential and limiting current. Pd/Ti*_n_*O_2*n*__–1_ has an onset potential of 90 mV, which is more negative than the Pt/C catalyst in the activation-controlled region, and the limiting current density is about 2.5 mA·cm^−2^. The 30% Pd/Vul and 30% Pd/KB catalysts show a similar behavior in terms of onset potential which is just slightly higher than that of Pd/Ti*_n_*O_2*n*__–1_. The diffusion-limiting current density of carbon-supported Pd catalysts (*j_d_*) reaches a value close to the commercial Pt catalyst. The *j_d_* is related to the number of electrons transferred per O_2_ molecule as reported in the Koutecky-Levich (K-L) equation:

(1) $$\frac{1}{j}=\frac{1}{j_{k}}+\frac{1}{j_{d}}=\frac{1}{j_{k}}+\frac{1}{0.62nFC_{O_{2}}D_{O_{2}}^{2/3}v^{-1/6}\omega^{1/2}}$$

where *j* is the overall current density; *j_K_* is the kinetic current density; *j_d_* is the diffusion current density; *n* is the number of electrons transferred in the ORR; *F* is the Faraday constant (96485 C·mol^−1^); $C_{{\text{O}}_{2}}$ (1.26 × 10^−3^ M) is the saturated concentration of oxygen; $D_{{\text{O}}_{2}}$ (2.60 × 10^−5^ cm^2^·s^−1^) is the diffusion coefficient of oxygen; *v* (9.00 × 10^−3^ cm^2^·s^−1^) is the kinematic viscosity of the solution, and *w* (100 rpm, 200 rpm, 400 rpm, 1000 rpm, 1600 rpm, 2500 rpm) is the electrode rotation rate. All the parameters, referred to the 0.1 M HClO_4_ solution, are reported in the literature [25]. As shown in Figure 4b, the K-L plots (*j*^−1^ *vs.* ω^−1/2^) of the ORR exhibited good linearity, indicating first-order kinetics with respect to the reactant concentration. The n value for Pd/KB and Pd/Vul, calculated from the slopes in the K-L plots, is 3.8. This indicates that oxygen reduction mainly involves a four-electron transfer pathway to produce water. The *n* value for Pd/Ti*_n_*O_2*n*–1_ is 2.1 in the same experimental conditions. This implies a reaction mechanism corresponding to the two-electron pathway which involves the reduction of oxygen to hydrogen peroxide. The lower *j_d_* of Pd/Ti*_n_*O_2*n*__–1_ is thus related to its lower number of electrons transferred in the ORR.

In Figure 5, a comparison of oxygen reduction reactions for the different catalysts in the presence of 0.1 M methanol in the bulk solution is shown. Under these conditions, the Pd/KB catalyst shows the most positive onset potential and reaches the largest limiting current density compared to that obtained with Pt/C. These results show the better tolerance to low methanol concentrations of Pd-based catalysts compared to a benchmark Pt catalyst.

KB used as a support material for Pd nanoparticles allows us to achieve a better distribution of Pd particles onto the support. The result is the obtainment of the smallest crystallite size among the synthesized supported catalysts. These aspects determine the highest ORR activity for the catalyst based on KB.

### 2.4. Accelerated Degradation Tests (ADTs)

**Figure 6:**
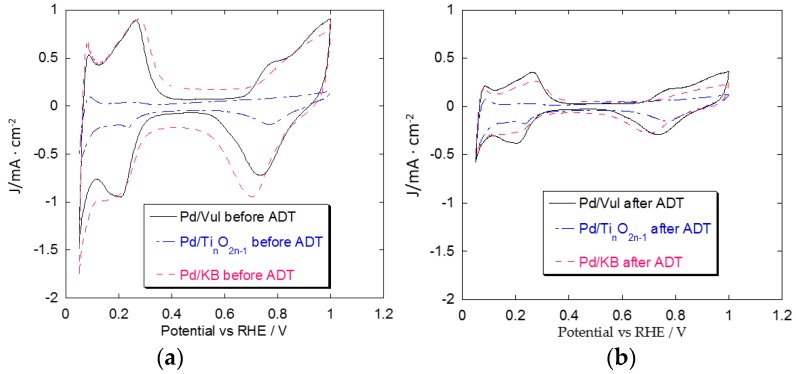
(**a**) CV of 30% Pd/Vul, 30% Pd/KB, 30% Pd/Ti*_n_*O_2*n*__–__1_ before ADT; (**b**) CV of 30% Pd/Vul, 30% Pd/KB, 30% Pd/Ti*_n_*O_2*n*__–__1_ after 1000 potential cycles.

The main kinetic parameters of Pd electrocatalysts are reported in Table 1. ECSA of Pd-based catalysts, before and after ADT, was determined in deaerated 0.1 M HClO_4_ using cyclic voltammetry (CV) (Figure 6a,b) by integrating the peak related to the Pd-oxide reduction between 0.4 and 0.8 V *vs.* RHE for Pd/KB and from 0.5 to 0.9 V *vs.* RHE for Pd/Vul and Pd/Ti*_n_*O_2*n*__–1_. A charge of 405 μC cm^−2^ for the reduction of a Pd-oxide monolayer was assumed according to the literature [26]. Among Pd/support catalysts, Pd/KB presents the highest ECSA before ADT. This is due to the higher specific surface area (in terms of BET) of the carbon support, leading to high dispersion and a lower Pd particle size (4.0 nm). After the ADT, Pd/KB shows the largest loss of ECSA (71%). Pd/Vul follows the same trend even if the loss of ECSA (60%) is slightly lower. Pd/Ti*_n_*O_2*n*__–1_ presents the smallest loss of ECSA (20%) after degradation. Such differences among carbonaceous and non-carbonaceous supports are also evident in the CVs (Figure 6) by comparing the profiles before and after ADT. The CV of the Ti*_n_*O_2*n*__–1_-supported Pd catalyst barely changes after ADT, while those related to Vul and KB present a significant variation.

**Table 1:** Kinetic parameters for the Pd/support catalysts.

Catalyst	ECSA/m^2^·g^−1^	Tafel Slope I Region/mV·dec^−1^	Tafel slope II Region/mV·dec^−1^	E_1/2_/V	SA at 0.85 V *vs.* RHE/mA·cm^−2^
*Before ADT*					
Pd/Vul	37.8	54.8	126.7	0.78	0.022
Pd/KB	39.0	63.5	143.0	0.80	0.033
Pd/Ti_n_O_2n__–__1_	9.10	48.8	126.9	0.79	0.056
*After ADT*					
Pd/Vul	15.2	48.6	133.5	0.77	0.054
Pd/KB	11.2	72.4	127.1	0.73	0.039
Pd/Ti_n_O_2n__–__1_	7.30	48.6	124.6	0.79	0.043

**Figure 7:**
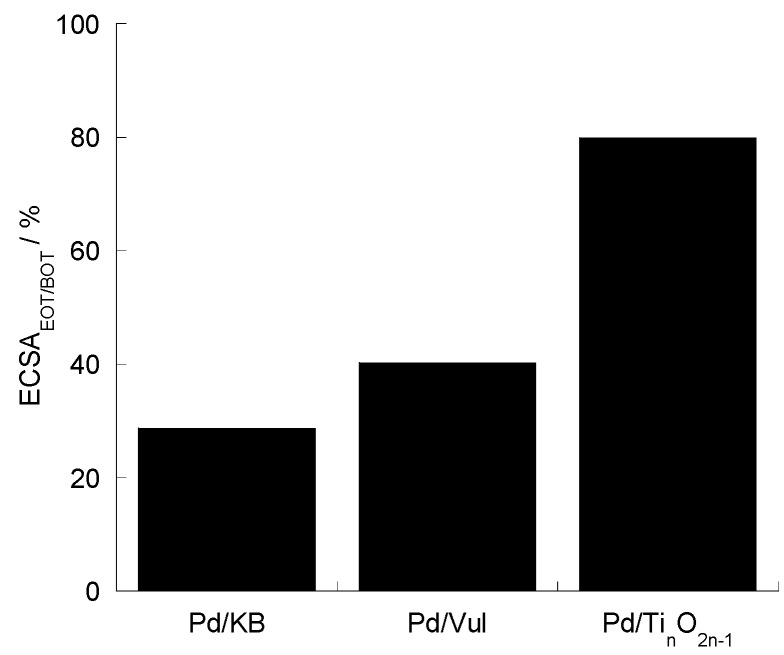
ECSA percentage for the different Pd/support catalysts obtained by the ratio between ECSA at EOT and BOT. Scan rate 5 mV·s^−1^; 1000 rpm; room temperature and 50 μg·cm^−2^ of Pd loading.

Figure 7 shows the percentage of the ECSA retention after ADT for the Pd catalysts. This is estimated from the ratio between the ECSA at the end of the test (EOT) and the same value at the beginning of the test (BOT). In light of the electrochemical results, the differences encountered in terms of ECSA decay can be ascribed to the different support surface area and composition, although the contribution of the initial Pd particle size cannot be discarded. The best results in terms of resistance to corrosion are obtained with the non-carbonaceous support (Ti-suboxides) [27].

The specific activity (SA) for the O_2_ reduction was calculated at 0.85 V *vs.* RHE after normalizing the kinetic currents by the catalyst loading and the ECSA (Table 1). The SA increases passing from carbon-supported Pd catalysts to Pd/Ti*_n_*O_2*n*__–1_. This appears to reflect the typical relationship observed for Pt catalysts where the occurrence of well-defined crystallographic orientations in large particles is associated with an increase of SA [28,29]. In contrast, small Pd particles contain a large number of defects which are less appropriate for a structure-sensitive reaction such as the oxygen electro-reduction. In addition to these effects, it may be possible that the metal support interaction is larger in carbon-supported samples where there is a significant occurrence of organic functional groups on the surface. The metal support interaction may decrease the number of Pd sites available for the chemisorption of oxygen.

**Figure 8:**
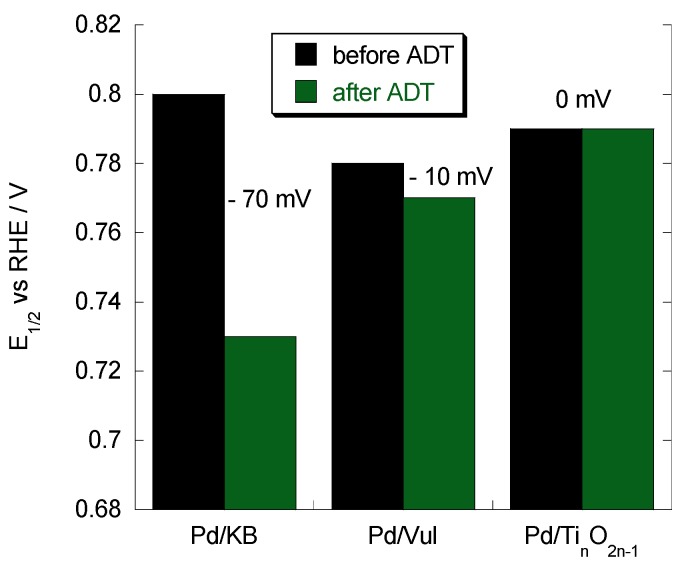
*E*_1/2_ of Pd/support catalysts before and after degradation cycles. Scan rate 5 mV·s^−1^; 1000 rpm; room temperature and 50 μg·cm^−2^ of Pd loading.

Pd catalysts present similar initial values of half-wave potential (*E*_1/2_), around 800 mV *vs.* RHE. The value of *E*_1/2_ for the Pd/Ti*_n_*O_2*n*__–1_ electrocatalyst does not change after 1000 potential cycles (Figure 8); however, it significantly decreases for carbonaceous support-based catalysts, especially for Pd/KB with a loss of 70 mV in *E*_1/2_. The values of the Tafel slope are also reported in Table 1, taking into account two regions: low current density (region I) and high current density (region II). There is no significant difference in terms of the Tafel slope produced by the support, and no changes have been observed for this slope after the ADT. This indicates that the oxygen adsorption mechanism is similar regardless of these variables. Kinetic parameters, reported in Table 1, are in agreement with the results previously reported in the literature [30,31].

**Figure 9:**
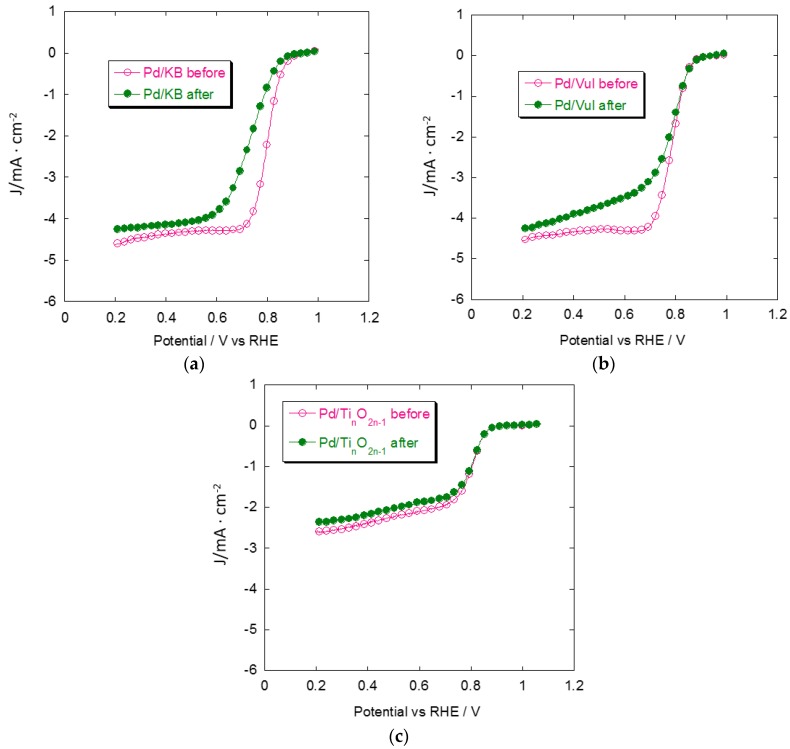
Oxygen reduction curves for the different Pd/support catalysts before and after degradation cycles. (**a**) Pd/KB; (**b**) Pd/Vul; (**c**) Pd/Ti*_n_*O_2*n*–1_. Scan rate 5 mV·s^−1^; 1000 rpm; room temperature and 50 μg·cm^−2^ of Pd loading.

Figure 9 shows the behavior of Pd catalysts for the ORR before and after the ADT. The activity of the Pd/Ti*_n_*O_2*n*__–1_ catalyst is almost unvaried, even after 1000 degradation cycles, indicating a very good stability of the non-carbonaceous support. It can be seen that Pd/KB loses significant activity after 1000 potential cycles both in kinetic and diffusion-limiting regions. Current density values for Pd/Vul, before and after ADT, present a similar trend in terms of onset and kinetic current but the loss of activity is pronounced below 0.8 V *vs.* RHE. These aspects can be mainly attributed to the difference between carbonaceous and non-carbonaceous support corrosion. The carbon blacks easily react with water to produce CO_2_ in the investigated potential region. In contract, Ti-suboxides show corrosion currents orders of magnitude lower than carbon blacks [27] and no clear electrochemical corrosion mechanism has yet been individuated in the literature [32,33].

These results highlight the advantage of using Pd/Ti*_n_*O_2*n*__–1_ in terms of resistance to corrosion phenomena. Further work to optimize Pd particle size would lead to high-performing cathode catalysts for application in DMFCs.

## 3. Materials and Methods

### 3.1. Pd-Based Electrocatalyst Preparation

First, 30% Pd supported on Vulcan-XC-72R (from Cabot, Boston, MA, USA), Ketjenblack (from AkzoNobel, Amsterdam, The Netherlands) and Ti suboxides (from Atraverda, Abertillery, Wales, UK) was prepared using the sulphite complex route [34]. An appropriate amount of support was ultrasonically dispersed in water for one hour and then mixed with Pd-sulphite acidic solution. Subsequently, H_2_O_2_ was added to decompose the sulphite complex with the formation of a colloidal suspension (PdO*_x_*/support) after pH correction (5.5). Hence, the suspension was filtered, copiously washed with water and dried at 80 °C. The obtained powder was carbothermally reduced at 500 °C in an inert atmosphere (Ar) to form Pd/Vul and Pd/KB. Pd/Ti*_n_*O_2*n*–1_ was formed by the reduction of the dried powder, at room temperature (25 °C), in a 10% H_2_–90% Ar atmosphere.

### 3.2. Physico-Chemical Characterization

X-ray diffraction (XRD) patterns for powder catalysts were recorded with a X’Pert 3710 X-Ray (Philips, Eindhoven, The Netherlands) diffractometer using a Cu-Kα source operating at 40 kV and 20 mA. The peak profiles of the (220) reflection for the face-centered cubic (fcc) structure of palladium or platinum phases were obtained by applying the Marquardt algorithm to calculate the crystallite size by the Debye-Sherrer equation. Instrumental broadening was determined by using a standard platinum sample.

Transmission electron microscopy (TEM) analysis was made by first dispersing the catalyst powder in isopropyl alcohol. A few drops of these solutions were deposited on carbon film-coated Cu grids and analyzed with a CM12 microscope (Philips, Eindhoven, The Netherlands). An amount of 200 Pd particles was counted to obtain a particle size distribution histogram for the carbon- or Ti-suboxides-supported catalyst.

The total metal content in the catalysts was determined by burning the carbon support in a thermal gravimetry experiment up to 950 °C in air (STA analyser, Netzsch, Bayern, Germany). The Pd content in Pd/Ti*_n_*O_2*n*–1_ catalyst was determined by energy dispersive X-ray (EDX) spectroscopy at 25 kV with an XL30 SFEG scanning electron microscope (FEI, Eindhoven, The Netherlands).

### 3.3. Electrochemical Measurements

All the electrochemical measurements of the catalysts were performed at room temperature using a conventional three-electrode cell. The reference electrode was an Hg/Hg_2_SO_4_ saturated with K_2_SO_4_. The counter electrode was a high-surface-area platinum wire and the working electrode was a glassy carbon (GC) disk of 5 mm where the thin film catalyst was deposited. The 30% Pd/support catalytic inks were prepared by sonicating each catalyst in isopropyl alcohol with 30% of Nafion for 30 min and then added onto a GC disk to obtain a 50 μg·cm^−2^ Pd loading. Before each measurement, the glassy carbon was polished with alumina suspension in an OP-Felt cloth. The commercial 30% Pt/C (from E-TEK) ink was prepared with the same procedure and used for comparison (same metal loading) with respect to supported Pd inks. The base electrolyte was a deaerated 0.1 M HClO_4_ solution in which methanol (MeOH) was gradually added to investigate the tolerance of the catalyst to the poisoning caused by this organic fuel at 50 mM and 100 mM MeOH concentrations. Conditioning of the catalyst was carried out, in the base electrolyte, by triangular voltage sweeps at a scan rate of 100 mV·s^−1^ between 0.05 and 1 V *vs.* reference hydrogen electrode (RHE). This procedure consisted of about 50 cycles up to obtaining a constant cyclic voltammogram (CV). Subsequently, three potential cycles were performed from 0.05 to 1 V *vs.* RHE, at a scan rate of 20 mV·s^−1^ to calculate the ECSA. ORR activity was evaluated by saturating the electrolyte with O_2_ and carrying out a linear sweep voltammetry from 1.1 to 0.2 V *vs.* RHE, at a scan rate of 5 mV·s^−1^. A rotating disk electrode (RDE) was used at different rotations speeds.

Accelerated degradation tests (ADTs) were performed by sweeping (1000 cycles) the potential from 0.6 to 1.0 V *vs.* RHE at a scan rate of 50 mV·s^−1^. The loss of ECSA and the other kinetic parameters were calculated before and after the stability tests in a deaerated 0.1 M HClO_4_ solution, whereas the loss of activity was evaluated with a linear sweep voltammetry in an oxygen-saturated electrolyte.

The electrochemical measurements were carried out with a Metrohm Autolab potentiostat/galvanostat.

## 4. Conclusions

The 30% Pd/Ketjenblack, 30% Pd/Vulcan and 30% Pd/Titanium suboxides have been synthesized and characterized for the oxygen reduction reaction to evaluate their activity in direct methanol fuel cell applications. It appears that Pd/Ketjenblack is the most active electrocatalyst in the presence of methanol in the base electrolyte, thus showing promising characteristics for direct methanol fuel cells. Instead, stability tests of these Pd-based catalysts indicate the best resistance to corrosion for the Ti-suboxide-supported Pd catalyst compared to carbonaceous supports, pointing out their good perspectives in terms of durability.
